# Giant right atrial thrombus unmasking Behçet’s disease: A rare cardiovascular manifestation

**DOI:** 10.1016/j.radcr.2026.08.047

**Published:** 2026-09-15

**Authors:** Houssam Ait Boutargante, Hicham Eddakhche, Amine El Houari, Wassim Beladel, Khalil Abderrahmane El Baz, Mehdi Berrajaa, Mohamed El Minaoui

**Affiliations:** Department of Cardiology, Mohammed VI University Hospital Center of Agadir, Faculty of Medicine and Pharmacy Ibn Zohr University, Agadir, Morocco

**Keywords:** Behçet’s disease, Right atrial thrombus, Intracardiac thrombosis

## Abstract

Behçet’s disease is a systemic vasculitis in which intracardiac thrombus formation is a rare but potentially life-threatening manifestation, predominantly involving the right heart. We report the case of a 37-year-old male presenting with pulmonary embolism associated with extensive central venous thrombosis and a giant right atrial thrombus, leading to the diagnosis of Behçet’s disease based on the 2014 International Criteria. Multimodality imaging using transthoracic echocardiography and contrast-enhanced computed tomography revealed a large heterogeneous right atrial thrombus and superior vena cava thrombosis. The patient was managed medically with a favorable evolution. Intracardiac thrombus should raise suspicion for Behçet’s disease in young patients with atypical venous thrombosis, as early diagnosis and immunosuppressive therapy are crucial for favorable outcomes.

## Introduction

Behçet’s disease is a chronic, multisystem vasculitis characterized by recurrent mucocutaneous ulcerations and variable vascular involvement [[1], [2], [3]]. While venous thrombosis is relatively common, intracardiac thrombus formation remains an exceptionally rare and severe complication, predominantly affecting the right heart chambers [[4], [5], [6]].

Early recognition through imaging is critical, as management requires prompt initiation of immunosuppressive therapy with or without anticoagulation [7].

We report a case of Behçet’s disease revealed by a giant right atrial thrombus associated with extensive central venous thrombosis, highlighting the diagnostic challenges and therapeutic considerations of this uncommon presentation.

## Case presentation

A 37-year-old man with recently diagnosed type 2 diabetes mellitus presented with chronic, painless cervical swelling of insidious onset. He denied constitutional symptoms or cardiovascular complaints, including dyspnea, chest pain, palpitations, or syncope.

On physical examination, multiple shallow oral ulcers and scrotal ulcerations consistent with bipolar aphthosis were observed, along with cutaneous lesions suggestive of pseudofolliculitis. There were no clinical signs of deep vein thrombosis in the extremities. Based on the 2014 International Criteria for Behçet’s Disease (ICBD), the diagnosis of Behçet’s disease was established [2].

Laboratory investigations demonstrated marked systemic inflammation, with leukocytosis (15 × 10⁹/L; reference range: 4-11 × 10^9^/L) and an elevated C-reactive protein concentration (54 mg/L; reference: 0-6 mg/L). Electrocardiography and chest radiography were unremarkable.

**Fig. 1:**
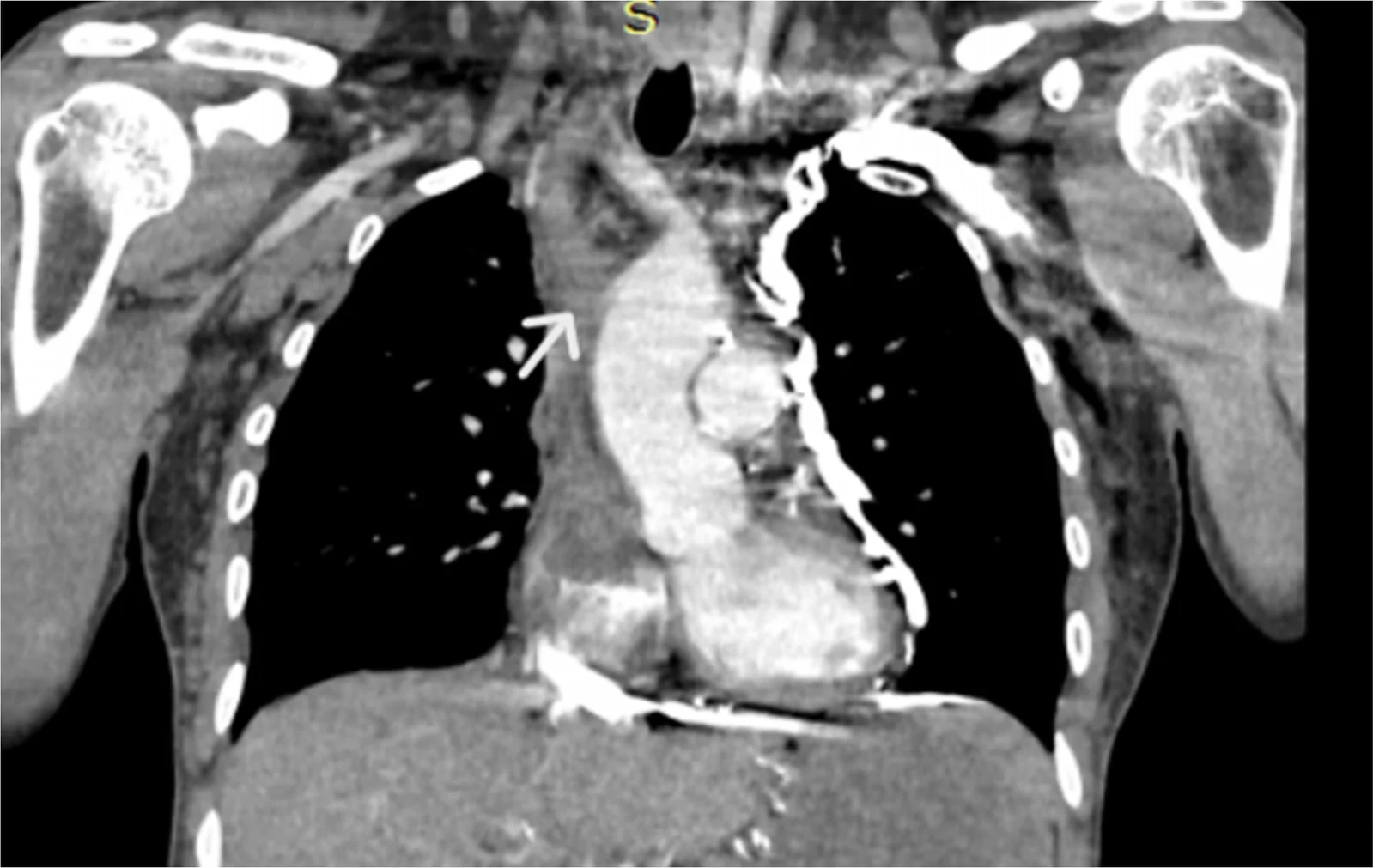
Coronal contrast-enhanced chest CT image showing a hypodense intraluminal filling defect (white arrow) within the superior vena cava (SVC), consistent with thrombotic occlusion. The superior vena cava appears partially obstructed, with extension of the thrombus along its course.

**Fig. 2:**
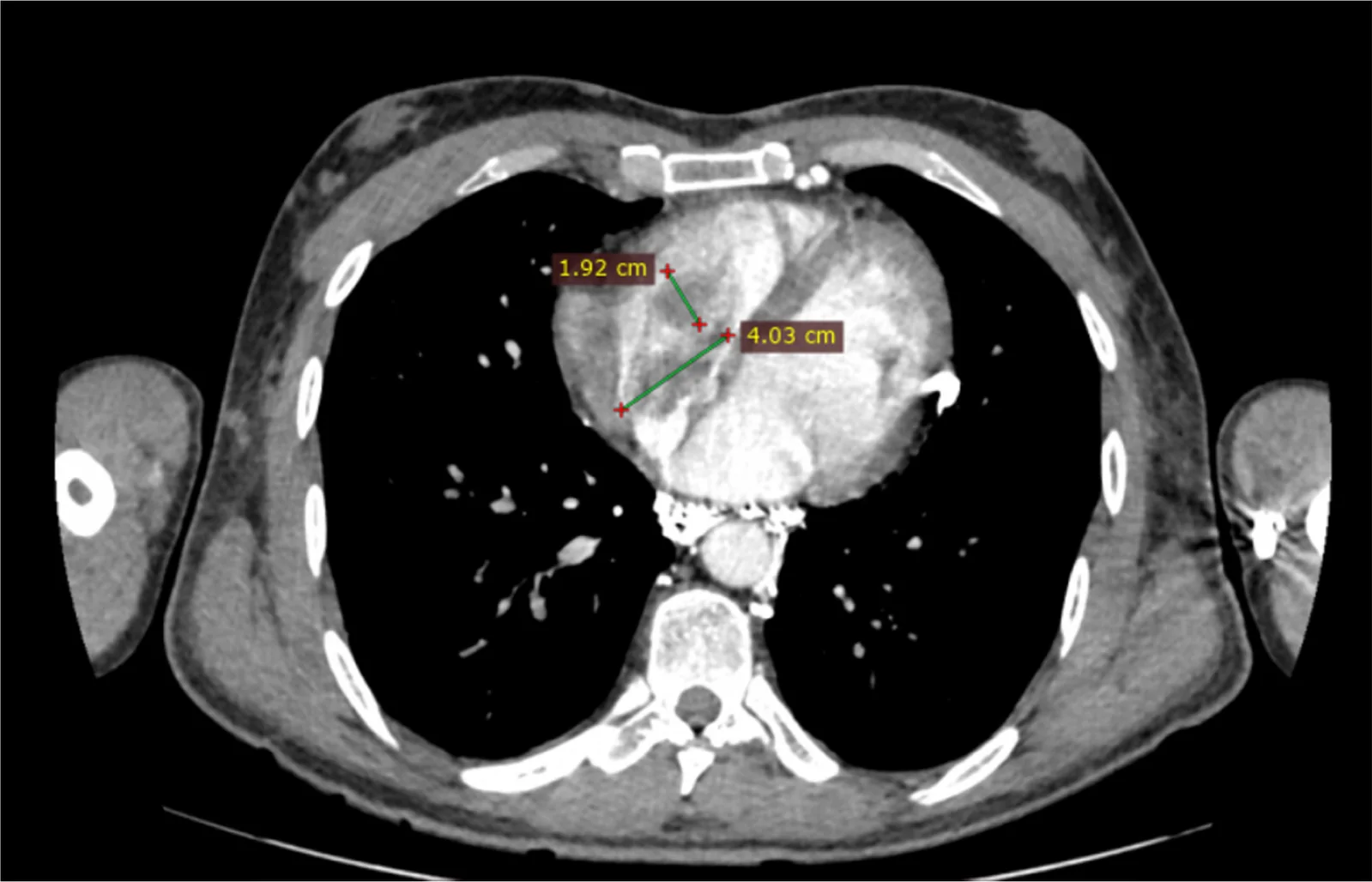
Contrast-enhanced axial chest CT image showing a large hypodense intracavitary filling defect within the right atrium (RA), consistent with thrombus. The lesion measures approximately 4.03 × 1.92 cm (calipers) and appears partially attached to the atrial wall.

**Fig. 3:**
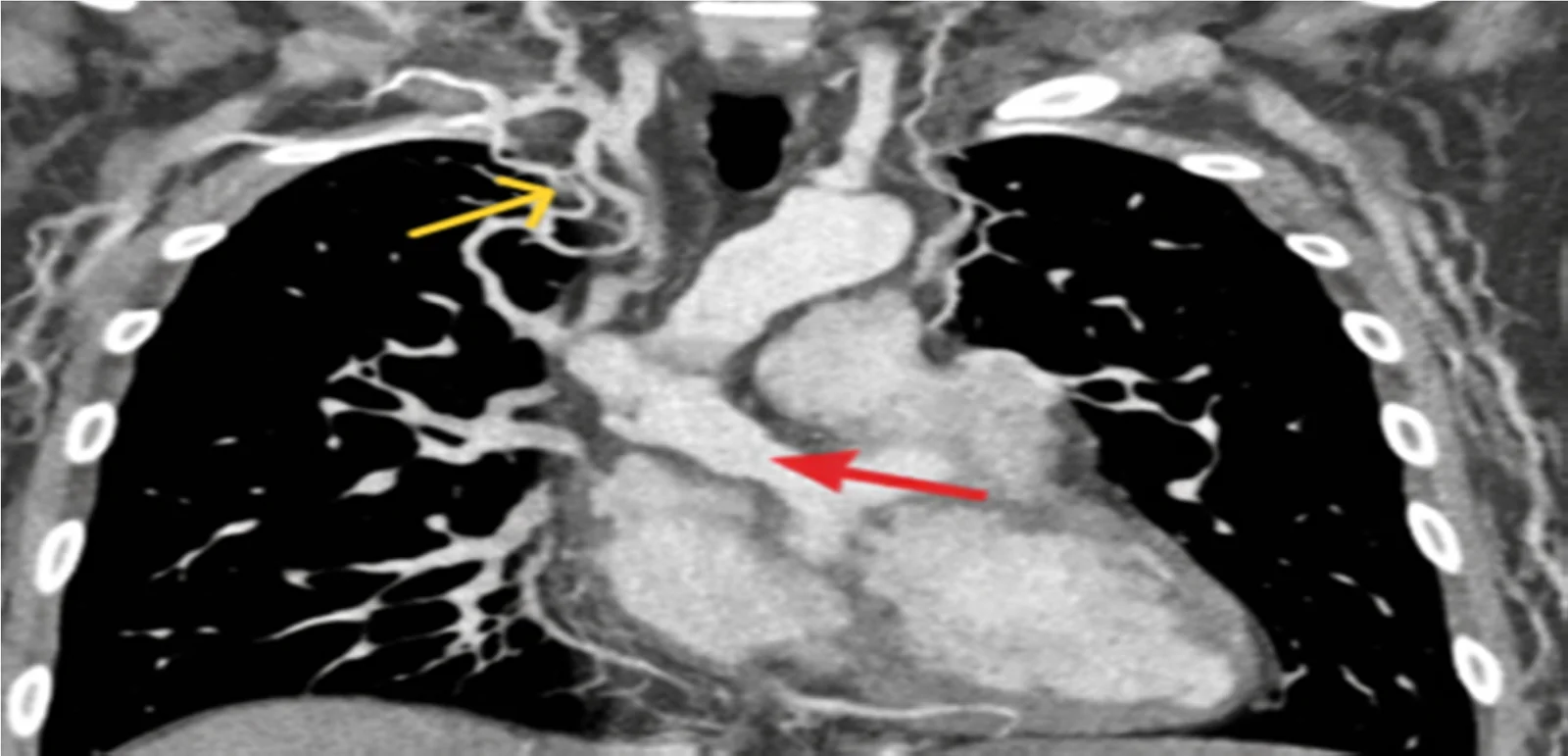
Coronal contrast-enhanced CT pulmonary angiography (CTPA) demonstrating a filling defect in the left lower lobar pulmonary artery (red arrow), consistent with pulmonary embolism. The image also shows prominent cervicothoracoabdominal collateral venous circulation (yellow arrow), reflecting chronic superior vena cava obstruction and extensive systemic venous rerouting.

Cervical ultrasonography revealed bilateral, well-defined laterocervical lymphadenopathies associated with perinodal fat stranding, findings consistent with reactive inflammatory changes. Contrast-enhanced thoracic computed tomography (CT) demonstrated chronic partial thrombosis extending from the superior vena cava into both brachiocephalic veins and the internal jugular veins bilaterally, accompanied by extensive cervicothoracoabdominal collateral venous circulation (Fig. 1). Additionally, a filling defect within the right atrium was identified, raising suspicion for an intracardiac thrombus (Fig. 2). The CT pulmonary angiogram (CTPA) confirmed a left lower lobar pulmonary embolism (Fig. 3). No pulmonary artery aneurysms or other arterial abnormalities were identified.

**Fig. 4:**
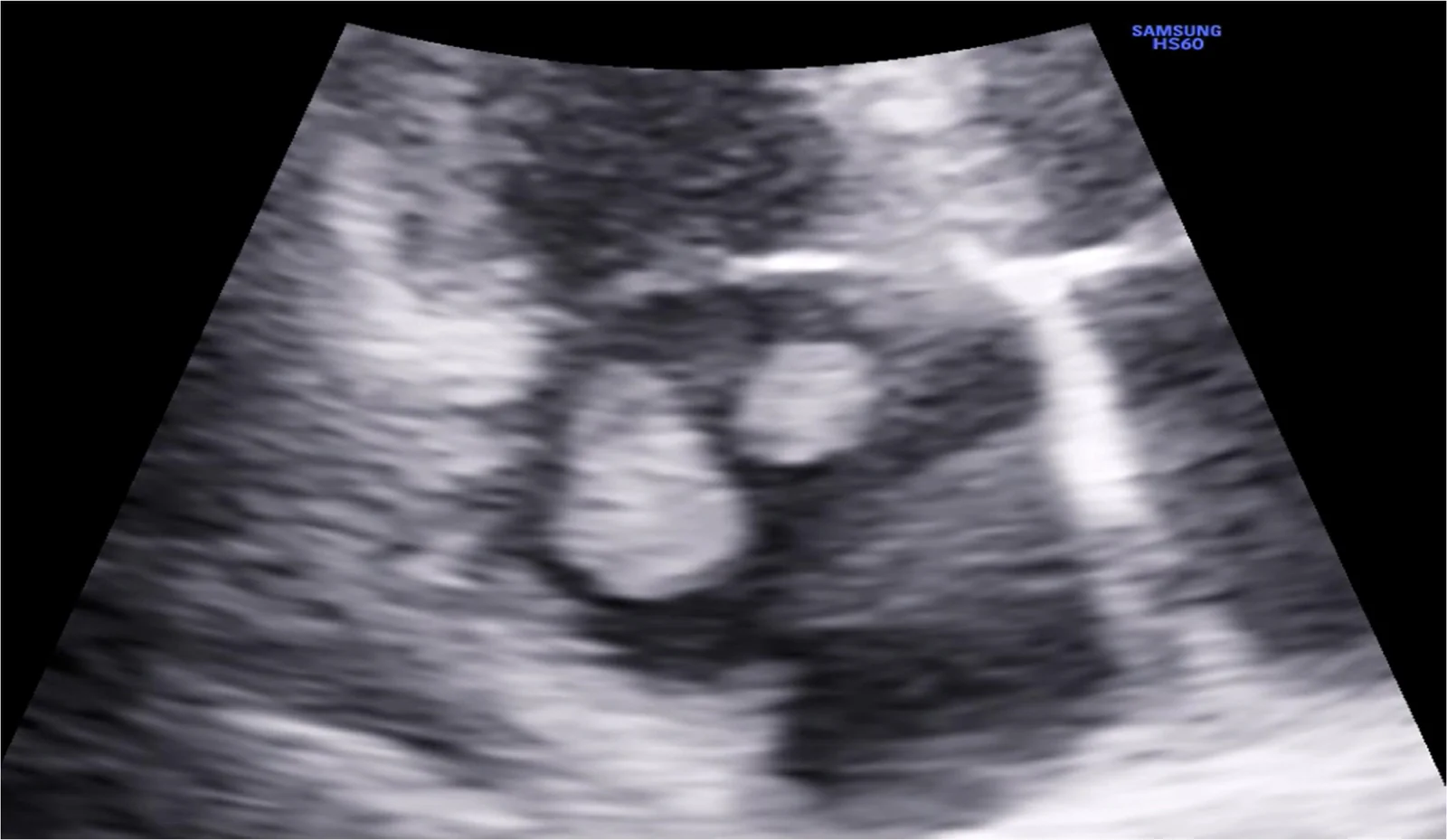
Focused transthoracic echocardiography (apical 4-chamber view) showing a heterogeneous, mobile echogenic mass within the right atrium (RA), protruding toward the tricuspid valve (TV), consistent with Behçet-related intracardiac thrombosis.

Transthoracic echocardiography confirmed the presence of a complex right atrial mass composed of 2 distinct components: a fixed, hyperechogenic segment adherent to the lateral atrial wall measuring 44 × 16 mm, and a mobile, multilobulated portion measuring 19 × 14 mm prolapsing into the atrial cavity (Fig. 4). These imaging features were highly suggestive of an organized intracardiac thrombus rather than an intracardiac tumor [4,8].

The patient was initiated on systemic corticosteroid therapy combined with monthly intravenous cyclophosphamide pulses [7]. Anticoagulation was introduced with low-molecular-weight heparin bridging to oral vitamin K antagonist (acenocoumarol), targeting an INR of 2.5.

**Fig. 5:**
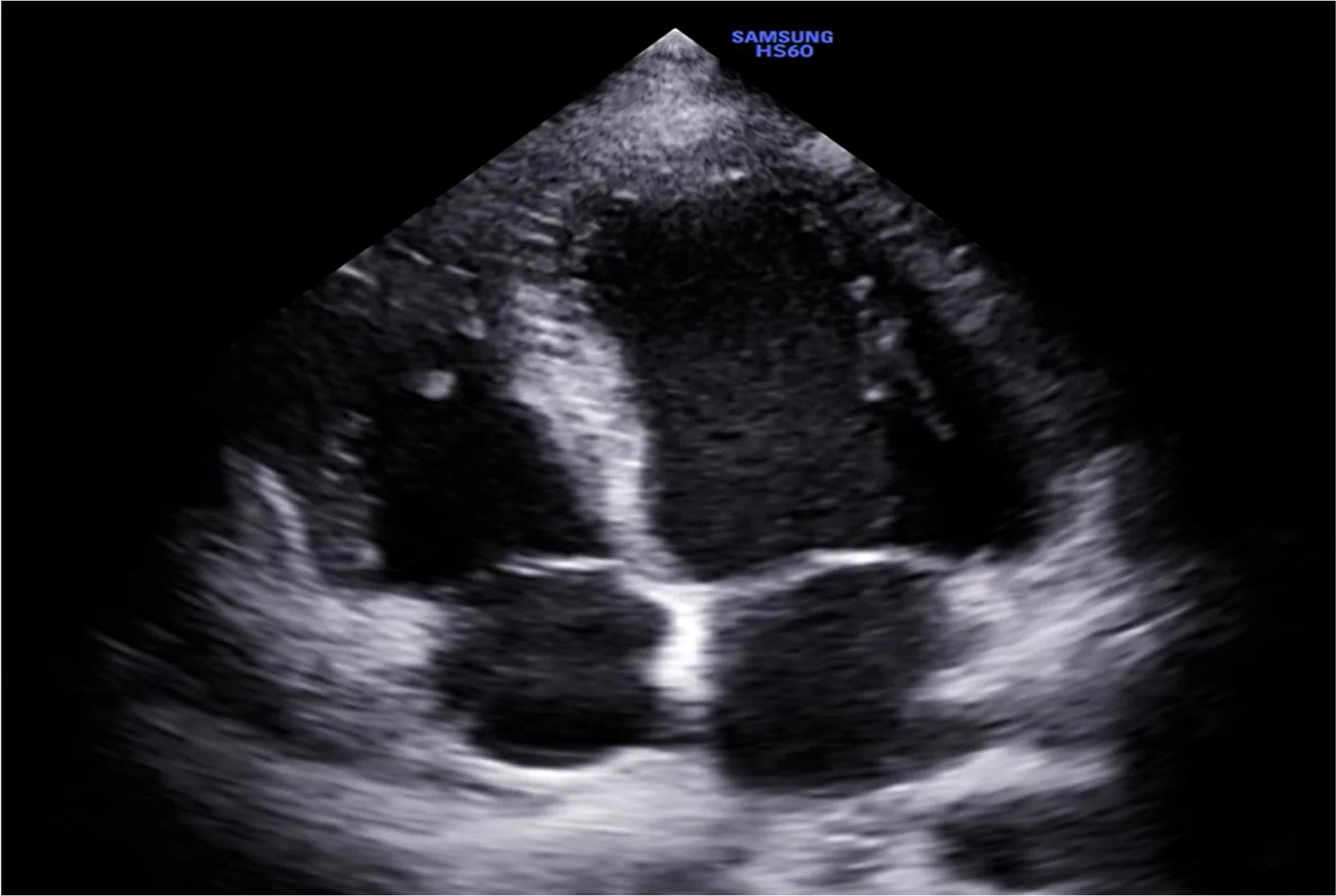
Follow-up transthoracic echocardiography (apical 4-chamber view) performed at 3 months showing complete resolution of the previously identified right atrial thrombus, with no residual intracavitary mass.

At 3-month follow-up, repeat transthoracic echocardiography and thoracic CT demonstrated complete resolution of the intracardiac thrombus and partial recanalization of the central venous system, with significant clinical improvement (Fig. 5).

## Discussion

Behçet’s disease is a rare, chronic, immune-mediated vasculitis that affects vessels of all sizes and types [1,3]. Its clinical expression is highly variable, with substantial interindividual differences in disease course, organ involvement, and response to treatment. Among its systemic manifestations, cardiovascular involvement remains relatively uncommon but carries a significant prognostic impact, with a reported mortality rate of up to 20% in cases with vascular complications [9,10].

Thrombotic events are the most frequent vascular manifestation of Behçet’s disease [3,7]. While deep vein thrombosis of the lower limbs is the most typical presentation, thromboses of atypical sites such as the superior vena cava, cerebral venous sinuses, pulmonary arteries, and even intracardiac chambers have been described [[4], [5], [6]]. Intracardiac thrombi are particularly rare, occurring in fewer than 2% of cases, and predominantly affect young men, typically involving the right heart cavities [4,5].

In the present case, the patient exhibited an unusual combination of chronic thrombosis of the superior vena cava and its major tributaries, a right atrial thrombus, and a left lower lobar pulmonary embolism. This constellation reflects the diffuse thrombotic potential of Behçet’s disease, even in the absence of traditional cardiovascular risk factors, and highlights the importance of systemic vascular imaging in evaluating unexplained venous occlusions, especially in young patients.

The pathogenesis of thrombosis in Behçet’s disease is multifactorial. Endothelial dysfunction due to immune-mediated inflammation, enhanced neutrophil activation, and a hypercoagulable state all contribute to thrombus formation [7]. Importantly, vascular involvement in Behçet’s disease is primarily driven by an inflammatory vasculitic process, and thrombotic events are often considered to arise in situ rather than from classical embolic mechanisms.

In this context, the origin of pulmonary artery involvement remains a matter of debate. While pulmonary embolism may theoretically result from fragmentation of an intracardiac thrombus or embolization from central venous thrombosis, several features in Behçet’s disease favor in situ pulmonary arterial thrombosis related to vasculitis. These include the strong inflammatory component of the disease, the tight adherence of thrombi to the vessel wall, and the relatively low incidence of embolic events despite extensive venous thrombosis.

In our patient, although an embolic contribution from the right atrial thrombus cannot be completely excluded, the overall clinical and pathophysiological context suggests that in situ pulmonary arterial thrombosis is the most likely mechanism.

The optimal management of intracardiac thrombus in Behçet’s disease remains controversial. While anticoagulation is a cornerstone of conventional thrombotic disease management, its role in Behçet’s is debated, primarily due to the risk of catastrophic hemorrhage in the presence of pulmonary artery aneurysms [7,10]—one of the most severe and potentially fatal complications of the disease. As such, current expert consensus favors prompt initiation of high-dose corticosteroids and immunosuppressive agents such as cyclophosphamide to target the underlying vasculitic process. Anticoagulation may be added in selected patients without evidence of pulmonary artery aneurysms and under close monitoring.

In our patient, the combination of immunosuppressive therapy and oral anticoagulation, initiated after careful exclusion of pulmonary arterial aneurysm, led to complete resolution of the intracardiac thrombus and clinical improvement without bleeding complications. This favorable outcome supports the use of combined therapy in well-selected cases and underscores the importance of individualized management guided by detailed imaging and multidisciplinary evaluation.

## Conclusion

Intracardiac thrombus is an exceptional but severe manifestation of Behçet’s disease, requiring a high index of suspicion, particularly in young patients with atypical or extensive venous thrombosis [[4], [5], [6]]. This case illustrates the importance of comprehensive vascular imaging to detect occult cardiac involvement and guide therapeutic decisions [8]. Early initiation of immunosuppressive therapy, with cautious use of anticoagulation in the absence of pulmonary artery aneurysms, can lead to complete thrombus resolution and favorable outcomes. Individualized, multidisciplinary management remains essential to optimize prognosis in this rare and complex presentation [7,10].

## Patient consent

Informed written consent was obtained from the patient for publication of the case report and all imaging studies.
